# Predictive and prognostic significance of tumour subtype, SSTR1‐5 and e‐cadherin expression in a well‐defined cohort of patients with acromegaly

**DOI:** 10.1111/jcmm.16173

**Published:** 2021-01-24

**Authors:** Jiri Soukup, Helena Hornychova, Monika Manethova, Kvetoslava Michalova, Ludmila Michnova, Lenka Popovska, Veronika Skarkova, Tomas Cesak, David Netuka, Ales Ryska, Jan Cap, Václav Hána, Václav Hána, Michal Kršek, Eva Dvořáková, Michal Krčma, Ivica Lazurova, Věra Olšovská, Karel Starý, Peter Vaňuga, Filip Gabalec

**Affiliations:** ^1^ The Fingerland Department of Pathology Faculty of Medicine University Hospital Charles University Hradec Kralove Czech Republic; ^2^ Department of Pathology Faculty of Medicine Charles University Plzen Czech Republic; ^3^ Bioptical Laboratory, Ltd. Plzen Czech Republic; ^4^ Department of Pathology Military University Hospital Prague Prague Czech Republic; ^5^ Department of Pharmacology and Toxicology Faculty of Pharmacy in Hradec Králové Charles University Hradec Kralove Czech Republic; ^6^ Department of Medical Biology and Genetics Faculty of Medicine Hradec Kralove Charles University Hradec Kralove Czech Republic; ^7^ Department of Neurosurgery Faculty of Medicine University Hospital Charles University Hradec Kralove Czech Republic; ^8^ Department of Neurosurgery and Neurooncology 1st Medical Faculty Charles University Military University Hospital Prague Prague Czech Republic; ^9^ 4th Department of Internal medicine Faculty of Medicine University Hospital Charles University Hradec Kralove Czech Republic; ^10^ 3rd Department of Internal Medicine 1st Faculty of Medicine Charles University and General University Hospital Prague Czech Republic; ^11^ 1st Department of Internal Medicine Faculty of Medicine in Pilsen University Hospital Pilsen Charles University Pilsen Czech Republic; ^12^ 1st Internal Clinic Louis Pasteur University Hospital Kosice Slovakia; ^13^ 2nd Department of Internal Medicine Faculty of Medicine St. Ann University Hospital Brno Masaryk University Brno Brno Czech Republic; ^14^ Department of Internal Medicine and Gastroenterology University Hospital Brno and Faculty of Medicine Masaryk University Brno Czech Republic; ^15^ National Institute of Endocrinology and Diabetology Lubochňa Slovakia

**Keywords:** acromegaly, PITNET, pituitary neoplasm, somatostatin receptor

## Abstract

In somatotroph pituitary tumours, somatostatin analogue (SSA) therapy outcomes vary throughout the studies. We performed an analysis of cohort of patients with acromegaly from the Czech registry to identify new prognostic and predictive factors. Clinical data of patients were collected, and complex immunohistochemical assessment of tumour samples was performed (SSTR1‐5, dopamine D2 receptor, E‐cadherin, AIP). The study included 110 patients. In 31, SSA treatment outcome was evaluated. Sparsely granulated tumours (SGST) differed from the other subtypes in expression of SSTR2A, SSTR3, SSTR5 and E‐cadherin and occurred more often in young. No other clinical differences were observed. Trouillas grading system showed association with age, tumour size and SSTR2A expression. Factors significantly associated with SSA treatment outcome included age, IGF1 levels, tumour size and expression of E‐cadherin and SSTR2A. In the group of SGST, poor SSA response was observed in younger patients with larger tumours, lower levels of SSTR2A and higher Ki67. We observed no relationship with expression of other proteins including AIP. No predictive value of E‐cadherin was observed when tumour subtype was considered**.** Multiple additional factors apart from SSTR2A expression can predict treatment outcome in patients with acromegaly.

## 1 INTRODUCTION

Pituitary neuroendocrine tumours (PitNETs) ^1^ producing growth hormone (GH) represent around 27% of all PitNETs^2^ and most of them manifest with acromegaly. Somatostatin analogue (SSA) treatment plays an important part in the therapy of PitNETs not cured using surgery alone.^3^, ^4^, ^5^ SSA therapy outcomes are variable throughout the studies in terms of achieving biochemical remission and significant tumour shrinkage.^4^, ^5^ Thus, there is a need for better identification of patients who would profit from SSA treatment. So far, somatostatin receptor 2A (SSTR2A) expression has significantly correlated with positive SSA response in most of the studies.^6^, ^7^, ^8^, ^9^ The importance of other somatostatin receptors (SSTRs) is not well understood. Among other factors that may modulate SSA treatment response, E‐cadherin and aryl hydrocarbon receptor‐interacting protein (AIP) have been studied repeatedly.^10^, ^11^, ^12^, ^13^ The prediction of SSA response and clinical behaviour of the tumour is important for the further clinical management of patients with acromegaly, and brings essential economic consequences as well. Thus, we decided to analyse the clinicopathological features of a large cohort of somatotroph PitNETs and to assess possible predictive factors in a subgroup of patients treated with SSA.

## 2 MATERIAL AND METHODS

### 2.1 Patients ‐ Cohort characteristics

In total, 293 patients with clinically and laboratory‐proven acromegaly caused by PitNET were identified in the Czechoslovak REgistry of SEllar Tumours (RESET). All patients were diagnosed between the years 2000 and 2015 and all underwent surgery. 115 samples were retrievable from the institutional archives, and 114 samples from 110 patients (54 women and 56 men) yielded enough material for analysis. Only samples from primary surgeries (n = 110) were used in the study. The data on IGF1 levels at presentation were available in 105 patients, GH levels in 104 patients (GH level as an average of 3 consecutive measurements during 1 hour), prolactin levels in 103 patients, TSH levels in 101 patients, the largest tumour diameter in 104 patients, tumour volume in 92 patients, and Knosp grade in 83 patients. Tumours with Knosp grade 0‐2 were considered non‐invasive, grades 3 and 4 were considered invasive. The mitotic count could be assessed in 102 tumours, p53 expression and the ‘A/B’ part of Trouillas grade in 107 tumours, and the overall Trouillas grade in 80 tumours. SSA treatment response in patients without concurrent radiotherapy could be assessed in 31 tumours. The response was evaluated as a percentage decrease of serum IGF1 levels during the period of SSA treatment. For the outcome analysis, patients were divided using both a 2‐tiered (good response – IGF1 reduction > 50%, and poor response ‐ IGF1 reduction < 50%) and a 4‐tiered system (IGF1 reduction < 20%; IGF1 reduction 20%‐50%; IGF1 reduction > 50% and attainment of normal IGF1 levels for age). The cut‐offs were defined arbitrarily. All the patients included in the RESET database had initially signed an informed consent agreeing with a future research use of the tissue. The design of the study was approved by the ethical committee of the first author's institution, where the experimental work was performed.

### 2.2 Immunohistochemistry

Archived formalin‐fixed paraffin‐embedded (FFPE) tissue blocks of tumours were collected from six participating institutions. Tissue blocks were cut into 4‐µm‐thick sections for routine H&E staining and additional immunohistochemical studies. The slides were reviewed for the presence of the PitNET. The list of antibodies and relevant details of immunohistochemistry protocols used for the study are summarized in Table S1. On slide positive controls were used. The sections staining was carried out on a Benchmark Ultra stainer from Ventana/Roche for most antibodies or on an Agilent/Dako Autostainer 48, using PT‐Link pretreatment system. All the techniques used for the visualization employed the avidin‐biotin complex method with horseradish peroxidase as the enzyme and DAB (3,3'‐diaminobenzidine) as the chromogen. All slides were subsequently counterstained with haematoxylin. Whereas all tumours were stained for prolactin, βTSH, cytokeratin 18, Ki67, p53, SSTR1, 2A, 3 and 5, E‐cadherin and D2 dopamine receptor (D2DR), only the tumours of patients treated with SSA were stained for AIP, and only tumours negative for cytokeratin 18 were stained with antibodies against cytokeratin 8/18, AE1/3, GH and GATA3.

### 2.3 PitNETs classification

All cases were reviewed by a single neuropathologist (JS). The percentage of cells positive for PRL and βTSH was recorded together with the cytokeratin 18 staining pattern and the percentage of cells containing fibrous bodies. The number of p53 strongly positive nuclei and the number of mitoses per 10 high power fields (HPF) was evaluated; strong staining in >10 nuclei for p53 was considered positive.^14^ The Ki67 index was counted in at least 500 cells in hot‐spots, the percentage was recorded, and ‘proliferative activity’ according to the previously published grading scheme was assessed.^14^ Where data on invasion were available, the overall Trouillas grade was assessed.

The tumours were subclassified according to the hormonal expression, cytokeratin 18 staining pattern, and overall morphology as suggested by current classification schemes (WHO and EPPG)^15^, ^16^:.tumours showing the presence of fibrous bodies in more than 70% of cells were classified as sparsely granulated somatotroph tumours (SGSTs), the remaining tumours were subclassifed according to the hormone expression into densely granulated somatotroph tumours (DGST), combined somatotroph and lactotroph tumours (SLTs), well‐differentiated plurihormonal tumours and plurihormonal Pit1^+^ tumours. Since the cohort was defined by the presence of acromegaly, we did not stain all the cases for GH as this would bring no additional information for the classificationpurposes. Similarly, we did not perform Pit1 immunohistochemistry as the positivity of all the included cases regardless of their hormonal profile would be expected, based on their lineage of origin.

### 2.4 SSTRs, E‐cadherin and D2DR immunohistochemistry evaluation

Evaluation of SSTR1‐5 and D2DR expression was performed by three observers (JS, MM and LP) independently after an initial consensus meeting at the multi‐head microscope. The percentage of positive tumour cells was established and the intensity of staining in the cellular subgroups was assessed on a scale from 1 to 3. The histoscore (H‐score) was then calculated for each tumour as the percentage of cells multiplied by their respective staining intensity, thus ranging from 0 to 300. Ie for a tumour with 30% of weakly positive cells, 20% of moderately positive cells and 10% of strongly positive cells, the H‐score was 100 (1*30 + 2*20 + 3*10). The pattern of positivity was recorded, and cells with other than membranous positivity were disregarded for evaluation of expression of SSTR2A and SSTR5. E‐cadherin and AIP positivity was evaluated in the same fashion by two independent observers (J.S, LP – E cadherin and JS, MM, ‐ AIP). Representative examples of immunohistochemical positivity for individual proteins are showed in supplementary Figure 1.

**Figure 1:**
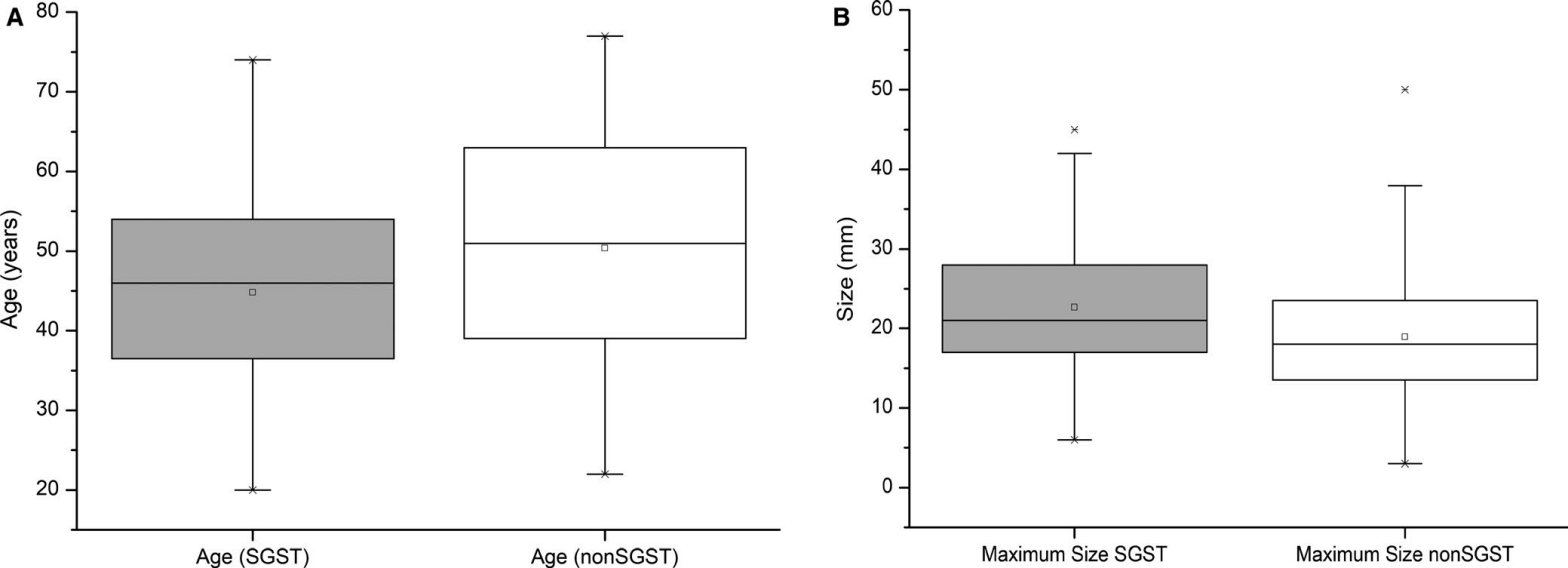
Sparsely granulated somatotroph tumours (SGST) showed statistically significant lower age and a tendency for bigger size compared to all non‐SGST histological subtypes of tumours

### 2.5 Statistical analysis

Statistical analysis was performed using SigmaPlot 14 software. Shapiro‐Wilk normality test was used to evaluate the distribution of the data. Two‐tailed variant of Student *t*‐test and one‐way analysis of variance (ANOVA) and their respective nonparametric versions, Mann‐Whitney test (MW) and Kruskal‐Wallis test (KW) were used to analyse continuous variables, along with χ^2^ and Fisher exact test to analyse categorical variables. Dunn test and Holm‐Sidak method were used for post hoc analysis. P‐values less than 0.05 were considered statistically significant.

## 3 RESULTS

### 3.1 Cases with preoperative treatment

Nine out of 110 patients received preoperative treatment with a first‐generation SSA. The median treatment duration before the surgery was 10 months. Compared to the untreated group, a significantly lower level of Ki67 index was observed in the pre‐treated group (median 0.79% *vs* 2.91%, *P* = .02, Mann‐Whitney test), while no difference in other parameters was found. In further analyses of Ki67, and Trouillas scoring system, we thus excluded pre‐treated cases.

### 3.2 Differences in histological subtypes

**Figure 2:**
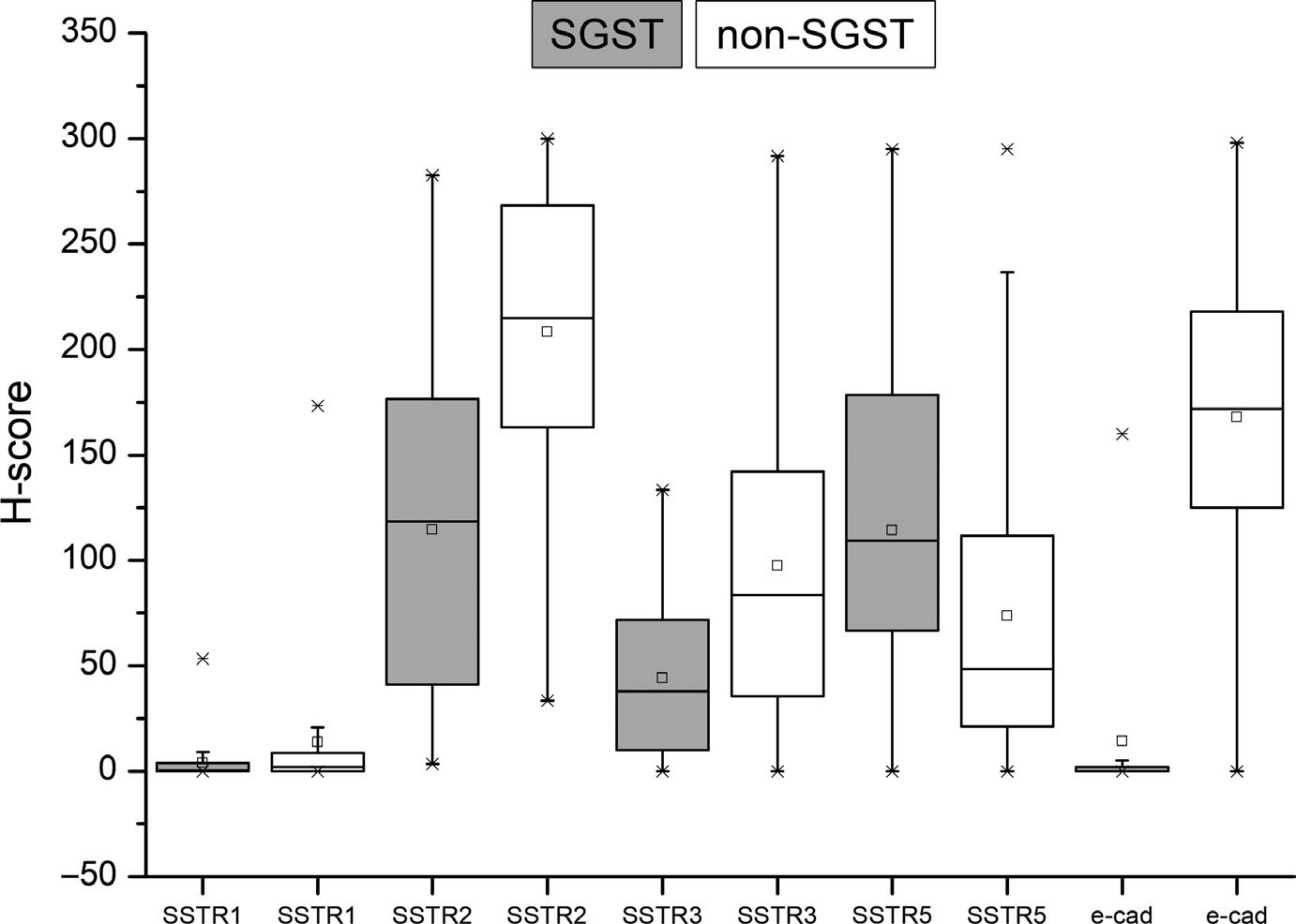
Sparsely granulated somatotroph tumours (SGST) compared to all non‐SGST histological subtypes of tumours showed significantly different expression of SSTR1‐3, SSTR5 and e‐cadherin

In total, we identified 45 SGST (41%, including 3 plurihormonal Pit1^+^ tumours), 29 DGST (26%), 26 SLT (24%) and 10 well‐differentiated plurihormonal tumours (9%). Nine tumours (8.2%) showed no expression of cytokeratins and these were classified according to their morphology and hormone expression. The only significantly different clinical feature among different tumour subtypes was younger age in the SGST subgroup compared to all non‐SGST tumours (DGST, SLT and plurihormonal groups) (44.73 ± 13.57 vs 50.59 ± 13.21 years; *P* = .026), and a slight trend for larger tumours (22.21 ± 9.69 vs 18.92 ± 8.80 mm, *P* = .07) – Figure 1. On the other hand, there was a significantly lower expression of E‐cadherin, SSTR1, SSTR2A, SSTR3 and higher expression of SSTR5 in the SGST subtype, compared to the other subtypes (Figure 2). Post hoc analysis of H‐scores showed significant differences between SGST and DGST (E‐cadherin, SSTR2A, SSTR3) and SGST and SLT (E‐cadherin, SSTR2A, SSTR5). At the same time, only E‐cadherin expression differed between SGST and plurihormonal tumours. Complete detailed characteristics of all histological subtypes are shown in supplemental Table S2.

### 3.3 ROC analysis

**Figure 3:**
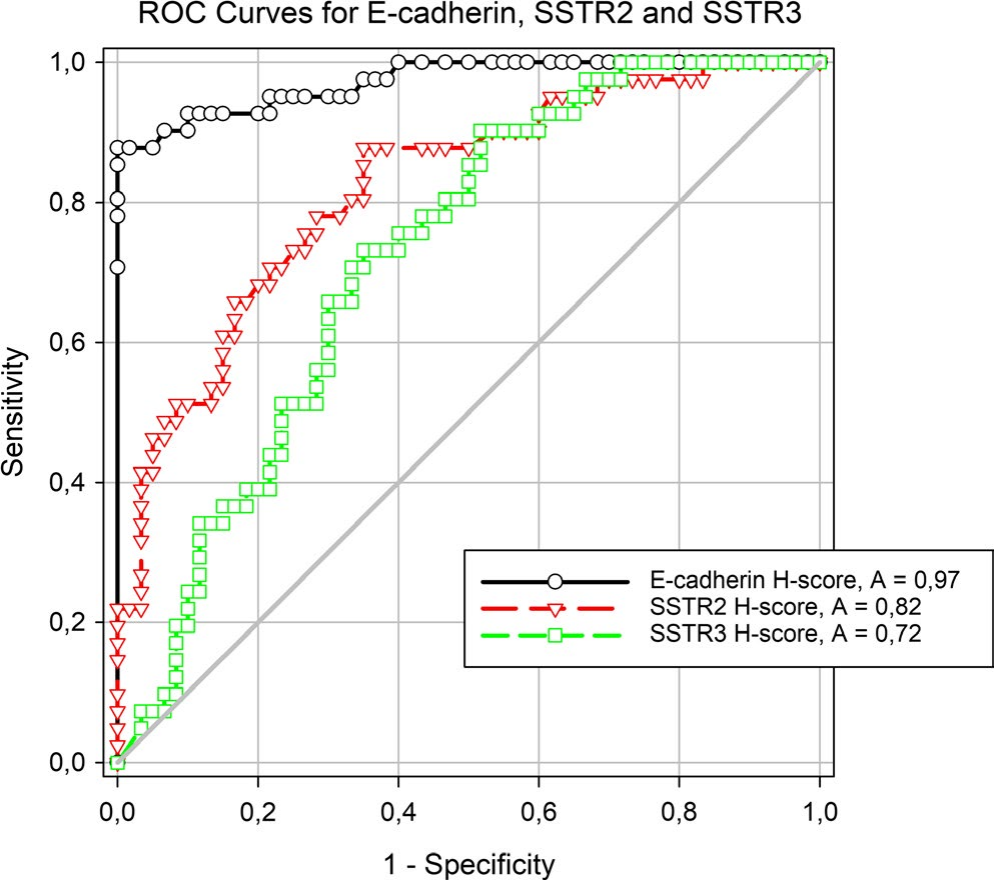
ROC curves for expression of E‐cadherin, SSTR2A and SSTR3 in SGST vs non‐SGST group as defined by cytokeratin expression; E‐cadherin was the most sensitive and specific feature of SGST (AUC = 0.97, *P* < .0001) followed by SSTR2A and SSTR3. Cytokeratin‐negative tumours were excluded from the analysis

Using ROC analysis in tumours defined by cytokeratin 18 expression (n = 101, Figure 3), low expression of E‐cadherin was the most sensitive and specific feature of SGST (AUC = 0.97, *P* < .0001) followed by SSTR2A (AUC = 0.82, *P* < .0001) and SSTR3 (AUC = 0.72, *P* = .0001). The difference between the AUC of E‐cadherin and SSTR2A was significant (*P* < .001).

### 3.4 Trouillas grade

Concerning the proliferative and non‐proliferative tumour subsets (Trouillas A and B), patients with proliferative tumours were significantly younger (40.77 ± SD 13.36 vs 52.70 ± SD 11.92, *P* < .001, Student *t*‐test) and showed higher GH levels (median 45.77 vs 26.16, *P* = .007, Mann‐Whitney test). Invasive tumours were significantly larger (mean 17.77 vs 22.91, *P* = .005, Student's *t*‐test) and showed significantly lower expression of D2DR (median 46.67 vs 14.17, *P* = .038, Mann‐Whitney test). When the complete Trouillas grade was considered, patients with grade 2b tumours were significantly younger compared to grades 1a and 1b (mean age 37.67, *P* = .003, ANOVA, Holm‐Sidak), they were significantly larger compared to 1a, 2a and 1b tumours (mean diameter 28 mm, *P* = .002, ANOVA, Holm‐Sidak), and showed significantly lower expression of SSTR2A (median 100, *P* = .048, Kruskal‐Wallis). No other clinical or pathological features differed.

### 3.5 Assessment of therapeutic response to SSA

**Figure 4:**
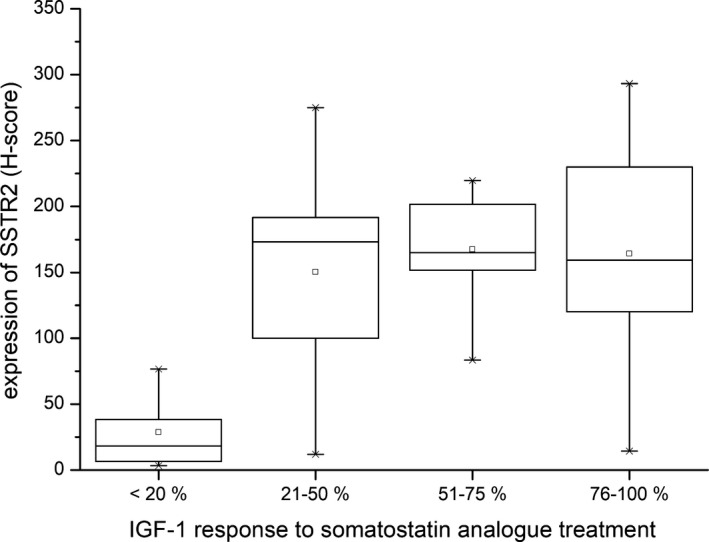
SSTR2A expression in somatotroph tumours according to the effect of the somatostatin analogue treatment. Mean expression of SSTR2A in Group 1 (patients with only small IGF‐1 < 20% reduction) was low and significantly different from the other groups (*P* < .001)

Treatment response after SSA administration could be assessed in 31 patients: these included 16 SGST, 9 SLT, 4 DGST, one well‐differentiated plurihormonal tumour and one plurihormonal Pit1^+^ tumour. Five patients from this group received concomitant treatment with cabergoline. Since these patients did not differ in outcome using either the 2‐tiered or 4‐tiered system (*P* = 1, Fisher exact test and *P* = .399, χ^2^ respectively), we decided to include them in the study. The median duration of treatment was 10.6 months in the good response group and 10.3 months in the poor response group. The number of patients in the different response subgroups and the medians of treatment duration are summarized in Table 1. A statistically significant positive correlation (Spearman correlation coefficient in the 4‐tiered subclassification) was found between treatment response and age (ρ = 0.39, *P* = .039), IGF1 level at the time of diagnosis (ρ = 0.37, *P* = .042), and SSTR2A expression (ρ = 0.39, *P* = .029). By contrast, the Ki67 index (ρ = −0.535, *P* = .007), serum TSH (ρ = −0.39, *P* = .033), the largest tumour diameter (ρ = −0.442, *P* = .015) and tumour volume (ρ = −0.419, *P* = .027) all correlated negatively with treatment response. Further comparison of the groups with good or poor prognosis (Table 2) showed significantly higher expression of SSTR2A and E‐cadherin and higher levels of IGF1 at the time of the diagnosis in the good responder group, while the group with poor response presented with significantly larger tumours and younger age. In the poor response group, only two patients belonged to the non‐SGST subset of tumours, and both showed levels of E‐cadherin comparable to the average and median of a good responder group. Using the 4‐tiered scale, the patients with no biochemical response (<20% reduction of IGF1) showed significantly lower H‐score of SSTR2A (*P* = .02, Kruskal‐Wallis) and larger tumour diameter (*P* = .025, one‐way ANOVA). The expression of somatostatin receptor SSTR2A in all groups is shown in Figure 4. The relationship between response and other parameters did not achieve statistical significance.

**Table 1:** A summary of treatment duration and response rates in patients treated with SSA

A summary of response rates is shown, along with median duration of treatment.			
	N°	N° (double‐step evaluation)	Treatment duration – median months
IGF1 reduction < 20%	5 (16%)	10 (32%)	13.3
IGF1 reduction 21‐50%	5 (16%)		7.2
IGF1 reduction > 50%	10 (32%)	21 (68%)	12.8
IGF1 normalization	11 (36%)		9.6

**Table 2:** Differences in clinical and histological parameters among tumours based on response to treatment

	Good response (±SD)	Insufficient response (±SD)	*P* value, test
Age (years)	47.27 ± 12.94	35.80 ± 15.52	***P* = .039 (Student´s *t*‐test)**
Serum GH (µg/l, n = 31)	63.08 ± 64.76	88.93 ± 70.88	*P* = .281 (MW)
Serum prolactin (µg/l, n = 31)	217.58 ± 732.68	314.613 ± 526.76	*P* = .06 (MW)
Serum TSH (mIU/l, n = 30)	1.06 ± 1.39	1 ± 0.29	*P* = .098 (MW)
Serum IGF1 (% above limit for the age, n = 31)	393.70 ± 155.90	271.70 ± 120.40	***P* = .038 (Student´s *t*‐test)**
Largest tumour diameter (mm, n = 30)	21.83 ± 8.25	31.58 ± 8.70	***P* = .006 (Student´s *t*‐test)**
Tumour volume (mm^3^, n = 28)	5379.61 ± 7121.07	11 090.89 ± 9326.60	***P* = .015 (MW)**
Prolactin immunoreactive cells (%)	25 ± 32.50	8.6 ± 8.92	*P* = .82 (MW)
bTSH immunoreactive cells (%)	0.3 ± 1.16	0 ± 0	*P* = .23 (MW)
Cells with fibrous bodies (%)	48 ± 41.70	80.5 ± 36.50	*P* = .07 (MW)
Ki67 index (%, n = 24)	3.03 ± 2.38	4.36 ± 0.893	*P* = .17 (Student´s *t*‐test)
Mitotic count (/10HPF, n = 28)	0.33 ± 0.77	0.9 ± 1.29	*P* = .25 (MW)
SSTR1 H‐score	15.87 ± 40.14	8.17 ± 17.67	*P* = .43 (MW)
SSTR2 H‐score	165.62 ± 66.44	89.53 ± 94.36	***P* = .015 (Student´s *t*‐test)**
SSTR3 H‐score	75.11 ± 53.35	47.16 ± 44.07	*P* = .13 (MW)
SSTR5 H‐score	88.64 ± 78.19	83.37 ± 49.16	*P* = .87 (MW)
D2DR H‐score	21.29 ± 21.34	22.9 ± 32.08	*P* = .81 (MW)
AIP H‐score	238.1 ± 37.16	227 ± 42.55	*P* = .464. Student´s *t*‐test
E‐cadherin H‐score	107.66 ± 95.51	26.6 ± 55.59	***P* = .03 (MW)**
Sex			
Men	14	3	
Women	7	7	*P* = .12 (Fisher´s exact test)
p53 Expression (n = 28)			
Positive	7	6	
Negative	11	4	*P* = .43 (Fisher´s exact test)
Proliferativity (n = 22)			
Trouillas A	12	2	
Trouillas B	3	5	*P* = .05 (Fisher´s exact test)
Invasivity (n = 23)			
Invasive	10	5	
Non‐invasive	7	1	*P* = .37 (Fisher´s exact test)

Bold indicate statistically significant values.

### 3.6 Analysis of the significance of the histological subtype for the response

The treated group included one cytokeratin‐negative tumour: we decided to exclude the patient from further analysis related to the tumour subtype due to the lack of literary data and consensus about subclassification of these tumours. Patients with SGST showed a trend towards poorer treatment response compared to patients from the non‐SGST group, but the finding did not achieve statistical significance (*P* = .058, Fisher's exact test).

### 3.7 Response to treatment in SGST patients

On a two‐step response scale, 8 patients showed a good response to treatment and 8 a poor response. Higher patient age (median 47.50 vs 29, *P* = .01, Mann‐Whitney test), a smaller tumour diameter (average 20.08 mm ± SD 6.94 vs 34.23 mm ± SD 7.55, *P* = .002, Student's *t*‐test),tumour volume (median 2227.7 mm^3^ vs 12 184.7 mm^3^ , *P* = .002, Mann‐Whitney test), lower proliferative activity evaluated by Ki67 index (average 2.039 ± SD 1.43 vs 4.36 ± SD 0.89, *P* = .004, Student's *t*‐test), and higher expression of SSTR2A (average 137.50 ± SD 76.93 vs 55.88 ± S.D 65.08, *P* = .038, Student's *t*‐test) all proved to be positive predictors of the therapeutic response in the SGST subgroup. The expression of E‐cadherin or AIP showed no significant difference between the two subgroups.

### 3.8 Response to treatment in patients with non‐SGST

There were 14 patients with non‐SGST altogether. Treatment response was superior to that in the SGST group: the reduction in IGF1 was in the range 20%‐50% in 2 patients (SLT and DGST), in 6 patients (3 DGST and 3 SLT) the reduction was by more than 50%, and in 6 patients the IGF1 level was fully normalized (5 SLT and 1 plurihormonal tumour). The distribution of treatment responses (poor response in 2 patients and good response in 12 patients) did not allow a meaningful statistical analysis, and hence, we decided to compare patients who achieved full normalization of IGF1 levels (n = 6) to patients who did not (n = 8). The only statistically significant factor by which the group with better therapeutic response differed was higher expression of SSTR5 (average 114.50 ± SD 57.73 vs 38.46 ± S.D 36.84, *P* = .011, Student's *t*‐test). No other clinical or pathological parameters, including E‐cadherin or AIP, achieved statistically significant predictive values.

## 4 DISCUSSION

We performed a comprehensive statistical analysis of the biological and clinical parameters of a large somatotroph PitNETs series and included the evaluation of potential predictors for treatment with a first‐generation SSA. The distribution of histological subtypes is comparable to the literature.^17^ However, our sample showed a higher prevalence of plurihormonal tumours: this might be due to the lack of standardized cut‐off values for the proportion of βTSH + cells that define this subgroup throughout the studies. When comparing clinical features of different pathological subsets, we observed only an association of SGST with younger age and a tendency for larger tumour size, when compared to non‐SGST. This is in accordance with other reports.^9^, ^18^, ^19^, ^20^ In terms of histological parameters, SGST and non‐SGST differed significantly in the expression of SSTR2, SSTR5 and E‐cadherin – findings consistent with the literature.^9^, ^21^, ^22^, ^23^, ^24^, ^25^

### 4.1 E‐cadherin

Low E‐cadherin expression in our study was the most sensitive and specific feature of SGST defined by the presence of fibrous bodies: in the non‐SGST group, only one case (1.5%) showed complete absence of E‐cadherin compared to 30 in the SGST group (71.4%). Three plurihormonal Pit1 + tumours showed absence of E‐cadherin as well; it is unknown whether this illustrates the general mechanisms (ie epithelial‐mesenchymal transition) responsible for the more aggressive behaviour of these tumours or reflects the possible relationship between the two entities, as suggested recently.^26^ The predictive value of E‐cadherin for SSA treatment has been suggested in two studies.^11^, ^12^ However, based on the results, we do not consider E‐cadherin to be an independent predictor, but rather a surrogate marker of the SGST subtype: this is illustrated by the fact that all the SGST in the poor response subgroup (8/10) showed no expression of E‐cadherin (H‐score 0) while two remaining non‐SGST tumours showed E‐cadherin expression comparable to the subgroup with good response (H‐scores 104 and 155). Moreover, we did not observe any differences in E‐cadherin scores between subsets of good and poor responders in SGST and non‐SGST subgroups.

### 4.2 SSTR3 and D2DR

The significantly higher expression of SSTR3 in non‐SGST has not been reported in the literature ^13^, ^22^, ^23^ and we interpret this finding as a result of more sensitive methodology (use monoclonal antibody UMB5 and H‐score instead of semiquantitative scales). We observed a significant association between low expression of D2DR and tumour invasion. Such finding has not been reported previously.^7^, ^27^, ^28^ However, due to the small difference in D2DR H‐score between the invasive and non‐invasive group, the clinical significance remains to be validated.

### 4.3 Trouillas grade

The relationship between Trouillas grade and the clinicopathological features of somatotrophs at the time of diagnosis has not been reported in the literature. In our study, tumours classified as 2b were significantly larger, showed lower expression of SSTR2A and were more common in younger patients.. A more aggressive phenotype of the disease has been previously reported in a subset of younger patients with SGSTs that showed higher Ki67 index.^29^ Although no association of Trouillas grade with histological subtype was observed in our study, we speculate that the agressive subset previously identified by the cluster analysis ^29^ and grade 2b tumours may at least partially overlap, given the phenotypic similarities. The reason for these findings is currently unknown, but it may reflect different pathogenesis of somatotroph tumours in different age groups.

### 4.4 Response to somatostatin analogue treatment – role of histological subtype and SSTR

Using the two‐tiered system, a good response to SSA was significantly associated with older age, higher IGF1 levels at the time of presentation, a smaller tumour size and higher expression of E‐cadherin and SSTR2A. Although the poor response was more common in SGST and Trouillas B tumours, statistical significance was not achieved. Importantly, SSTR2A was significantly lower in all the patients showing no response to SSA (IGF1 reduction < 20%) compared to the rest. SSAs of the first generation show the highest affinity for this receptor, and it is generally considered the most important for SSA signalling in the therapeutic context.^30^ Similar results were observed in most of the other studies.^7^, ^8^, ^9^, ^31^, ^32^ SGSTs have been associated with poor SSA response in the literature previously.^9^, ^33^ However, a good response was achieved in 50% of SGST in our cohort, and predictors of good response for SGSTs were the higher expression of SSTR2A, older age, smaller size of tumour, and lower Ki67 index. The other SSTR subtypes did not play a role in predicting the response in our study, with the exception of SSTR5 in the non‐SGST subgroup: patients with higher SSTR5 expression achieved biochemical remission significantly more often compared to the rest of the group. A possible explanation might be dimerization of the two receptor subtypes.^34^ SSTR2A and SSTR5 can form heterodimers, and this enhances the functionality of the SSTR2A signalling pathway and increases membrane recirculation of the receptors after previous internalization.^35^

### 4.5 Response to somatostatin analogue treatment – role of AIP

Recently, low expression of AIP in sporadic somatotroph tumours was also associated with poor response to the first‐generation SSAs,^10^, ^13^, ^31^ higher Ki67 index,^10^ larger tumour size at presentation, and SGST subtype.^36^ In our study, however, we observed no association between AIP H‐score and SSA response or any other clinicopathological parameters, including histological subtypes. In our work, we observed levels of H‐score for AIP similar to those from the largest available study on the subject.^31^ Using the same antibody (clone 35‐2) at 5‐times greater dilution, the median H‐score for AIP was 230 and 240 in our study, compared to 220 and 270 in the poor response and good response groups of the aforementioned paper. While the lack of association between AIP and SSA response has also been reported previously,^36^ this discrepancy may merely reflect methodological difficulties in immunohistochemical detection and interpretation of AIP expression. According to The Human Protein Atlas,^37^, ^38^ AIP is ubiquitously expressed in high quantities throughout the human tissues, and currently, there are no methodological guidelines on how to calibrate the immunohistochemical assay. This may hinder the reproducibility of immunohistochemical detection of AIP in the future and hamper routine clinical use.

## 5 CONCLUSION

It is important to subclassify somatotroph PitNETS correctly according to their histological features because the individual tumour subtypes may differ in biological and clinical features. Although a poor response to SSA is more common in the SGST subtype, the subgroup is not homogeneous with respect to treatment response, and different predictive factors play a role in this context. Low SSTR2A receptor expression is significantly associated with a low response to treatment with a somatostatin analogue regardless of histological subtype. Although low E‐cadherin expression is a predictor of poor outcome, we were unable to prove its value independent of the histological subtype of tumour, and in our opinion, it is merely a characteristic SGSTs, which in general tend to show more unsatisfactory response. We were unable to show a predictive value for AIP expression, possibly due to the lack of standardization of the immunohistochemical reaction, and this may limit the routine use of the antibody in this context. At least in non‐SGSTs, expression of SSTR5 might play an advantageous role in attaining a biochemical response.

## CONFLICT OF INTEREST

The authors declare no conflicts of interest.

## AUTHOR CONTRIBUTIONS

**Jiri Soukup:** Data curation (equal); Formal analysis (equal); Investigation (lead); Methodology (equal); Visualization (equal); Writing‐original draft (equal); Writing‐review & editing (equal). **Helena Hornychova:** Investigation (supporting); Methodology (lead). **Monika Manethova:** Formal analysis (equal); Investigation (equal); Methodology (supporting). **Květoslava Michalová:** Data curation (equal). **Ludmila Michnová:** Data curation (equal). **Lenka Popovská:** Formal analysis (equal); Investigation (equal). **Veronika Skarková:** Methodology (equal); Validation (equal). **Tomas Cesak:** Data curation (equal). **David Netuka:** Data curation (equal). **Ales Ryska:** Supervision (equal); Writing‐review & editing (equal). **Jan Cap:** Supervision (equal); Writing‐review & editing (equal). **Václav Hána:** Data curation (equal); Writing‐review & editing (equal). **Václav Hána, Jr:** Data curation (equal); Writing‐review & editing (equal). **Michal Krsek:** Data curation (equal); Writing‐review & editing (equal). **Eva Dvorakova:** Data curation (equal). **Michal Krcma:** Data curation (equal). **Ivica Lazurova:** Data curation (equal); Writing‐review & editing (equal). **Vera Olsovska:** Data curation (equal). **Karel Stary:** Data curation (equal). **Peter Vanuga:** Data curation (equal). **Filip Gabalec:** Conceptualization (lead); Data curation (equal); Formal analysis (equal); Funding acquisition (lead); Project administration (lead); Writing‐original draft (equal); Writing‐review & editing (equal).

## Supporting information

Fig S1

Table S1

Table S2
