# Supplementary figures and images for: Predictive and prognostic significance of tumour subtype, SSTR1‐5 and e‐cadherin expression in a well‐defined cohort of patients with acromegaly
Source: J Cell Mol Med. 2021 Jan 24;25(5):2484–92. doi: 10.1111/jcmm.16173 (PMC7933931; doi:10.1111/jcmm.16173)

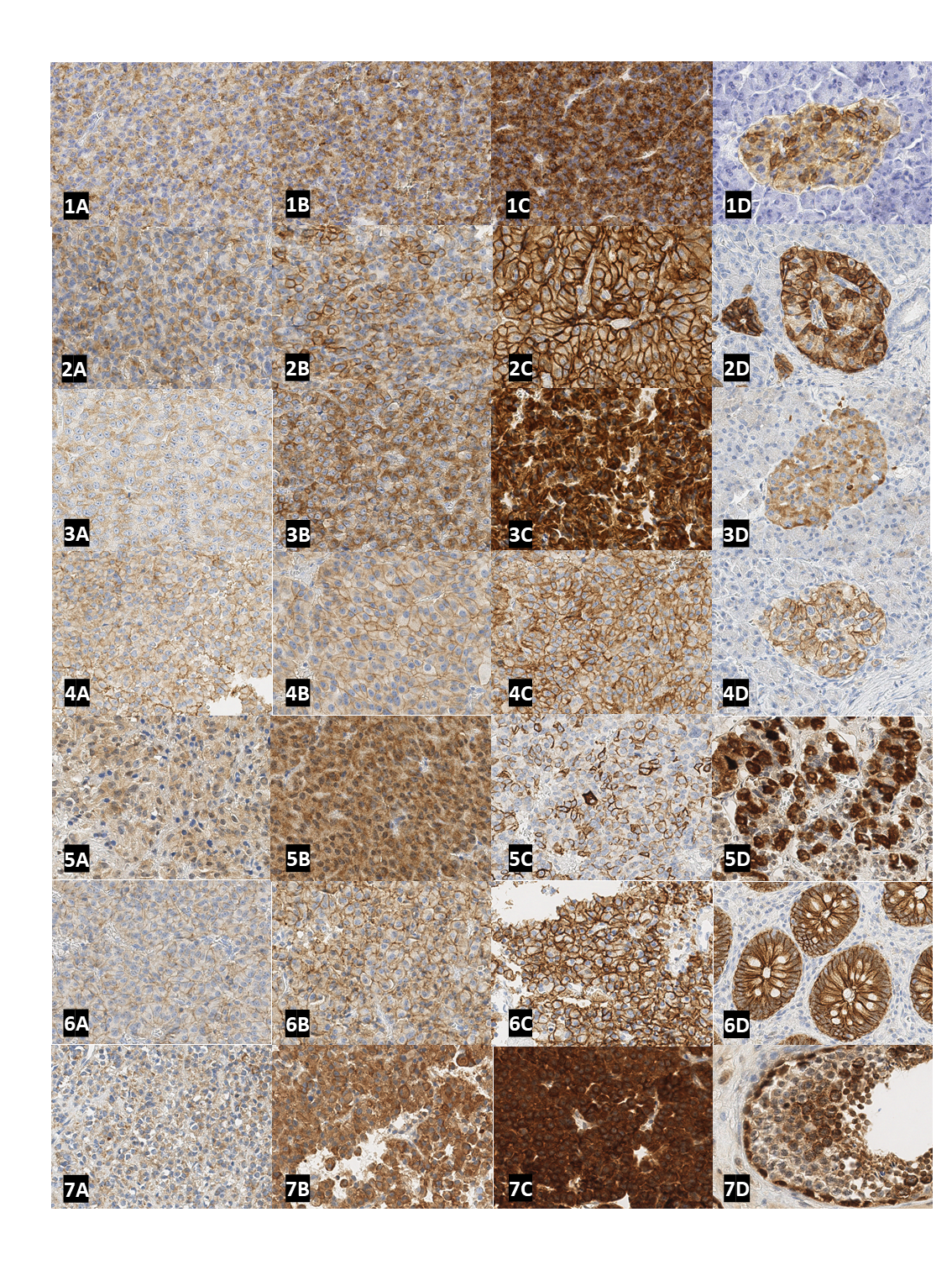

Supplement: Supplementary file 1 — Fig S1 [file JCMM-25-2484-s001.tif]
