# Supplementary material for: Predictive and prognostic significance of tumour subtype, SSTR1‐5 and e‐cadherin expression in a well‐defined cohort of patients with acromegaly
Source: J Cell Mol Med. 2021 Jan 24;25(5):2484–92. doi: 10.1111/jcmm.16173 (PMC7933931; doi:10.1111/jcmm.16173)
Supplement: Supplementary file 2 — Table S1 [file JCMM-25-2484-s002.pdf]

Antibody	Clone	Dilution	Manufacturer	Pretreatment	Incubation time	Detection system	Positive control
Prolactin	polyclonal	RTU	Ventana, Basel, Switzerland	Ventana CC1, 64 min.	48 minutes	Ventana ultraView	Normal pituitary
STH	EP267	1:200	Cell Marque, Rocklin, CA, USA	Ventana CC1, 32 min.	32 minutes	Ventana OptiView	Normal pituitary
βTSH	polyclonal	RTU	Ventana, Basel, Switzerland	Ventana CC1, 24 min.	32 minutes	Ventana ultraView	Normal pituitary
P53	Bp53-11	RTU	Ventana, Basel, Switzerland	Ventana CC1, 64 min.	24 minutes	Ventana ultraView	Breast carcinoma
Cytokeratin 18	DC10	1:50	Dako, Glostrup, Denmark A/S	Ventana CC1, 36 min.	32 minutes	Ventana ultraView	Normal appendix
Cytokeratin 8/18	B22.1/B23.1	1:200	Cell Marque, Rocklin, CA, USA	Ventana CC1, 48 min.	32 minutes	Ventana OptiView	Normal liver
Cytokeratin AE1/3	AE1/3	1:50	Dako, Glostrup, Denmark A/S	Ventana CC1, 64 min.	32 minutes	Ventana OptiView	Normal kidney
GATA3	L50-823	1:500	Cell Marque, Rocklin, CA, USA	Ventana CC1, 32 min.	36 minutes	Ventana OptiView	Normal kidney
Ki67	30-9	RTU	Ventana, Basel, Switzerland	Ventana CC1, 32 min.	16 minutes	Ventana OptiView	Normal appendix
E-cadherin	NCH-38	1:50	Dako, Glostrup, Denmark A/S	Ventana CC1, 52 min.	32 minutes	Ventana ultraView	Normal appendix
SSTR1	UMB7	1:100	Abcam, Cambridge, MA, USA	EnVision FLEX High pH, 97°C, 20 min.	30 minutes	DAKO EnVision FLEX	Endocrine pancreas
SSTR2A	UMB1	1:1500	Abcam, Cambridge, MA, USA	Ventana CC1, 36 min.	32 minutes	Ventana ultraView	Endocrine pancreas
SSTR3	UMB5	1:750	Abcam, Cambridge, MA, USA	Ventana CC1, 20 min.	36 minutes	Ventana ultraView	Endocrine pancreas
SSTR5	UMB4	1:750	Abcam, Cambridge, MA, USA	Ventana CC1, 32 min.	32 minutes	Ventana OptiView	Endocrine pancreas
D2DR	polyclonal	1:200	Origene, Rockville, MD, USA	Ventana CC1, 16 min.	32 minutes	Ventana OptiView	Normal pituitary
AIP	35-2	1:2500	Novus Biologicals, Centennial, CO, USA	Ventana CC1, 36 min.	36 minutes	Ventana ultraView	Normal testis
