# Supplementary material for: Predictive and prognostic significance of tumour subtype, SSTR1‐5 and e‐cadherin expression in a well‐defined cohort of patients with acromegaly
Source: J Cell Mol Med. 2021 Jan 24;25(5):2484–92. doi: 10.1111/jcmm.16173 (PMC7933931; doi:10.1111/jcmm.16173)
Supplement: Supplementary file 3 — Table S2 [file JCMM-25-2484-s003.docx]

|  | DGST (n=29) | SLT (n=26) | Plurihormonal tumours (n=10) | SGST (n=45) | P value, test | Non-SGST (n=65) | P value, test (SGST vs non-SGST) |
| --- | --- | --- | --- | --- | --- | --- | --- |
| Age (years) | 51.10±14.52 | 48.65±12.73 | 54.10±10.47 | 44.73±13.57 | P=0.10 (ANOVA) | 50.59±13.21 | **P=0.026 (Student's t-test)** |
| Serum GH (µg/l, n=104) | 61.39±74.22 | 55.69±56.79 | 54.13±87.24 | 47.64±52.77 | P=0.39 (KW) | 57.92±68.92 | P=0.32 (MW) |
| Serum prolactin (µg/l, n=103) | 44.47±85.89 | 90.33±200.44 | 281.19±777.18 | 221.65±605.49 | P=0.61 (KW) | 101.88±330.62 | P=0.84 (MW) |
| Serum TSH (mIU/l, n=101) | 1.08±0.64 | 1.01±0.73 | 1.4±1.09 | 1.13±1.04 | P=0.74 (KW) | 1.1±0.76 | P=0.88 (MW) |
| Serum IGF1 (% above limit for the age, n=105) | 314.1±122.10 | 310.5±109.20 | 332.4±231.10 | 307±119.80 | P=0.86 (KW) | 315.5±137.60 | P=0.89 (MW) |
| Largest tumour diameter (mm, n=104) | 20.37±10.59 | 17.88±6.36 | 17.6±9.06 | 22.21±9.69 | P=0.31 (KW) | 18.92±8.80 | P=0.07 (MW) |
| Tumour volume (mm^3^, n=92) | 6519.38±10457.91 | 2060.33±1909.53 | 3515.04±3553.59 | 5414.42±7258.03 | P=0.21 (KW) | 4151.49±7177.68 | P=0.07 (MW) |
| Prolactin immunoreactive cells (%) | 4.72±14.70 | 45.8±28.30 | 20±19.9 | 14.7±21.60 | **P <0.001 (KW)** | 23.5±28.70 | P=0.29 (MW) |
| bTSH immunoreactive cells (%) | 0.01±0.31 | 0.1±0.33 | 16.5±20.5 | 1.22±4.09 | **P <0.001 (KW)** | 2.63±9.74 | P=0.69 (MW) |
| Cells with fibrous bodies (%) | 4.59±9.17 | 25.6±22 | 18.50±22.7 | 89.3±21 | **P <0.001 (KW)** | 15.3±20 | **P<0.001 (MW)** |
| Ki67 index (%, n=101) | 2.6±1.99 | 3.45±1.76 | 3.26±1.65 | 3.47±2.03 | P = 0.20 (KW) | 3.05±1.86 | P=0.31 (MW) |
| Mitotic count (/10HPF, n=102) | 0.29±0.54 | 0.56±1.19 | 0.30±0.68 | 0.63±0.63 | P = 0.36 (KW) | 0.4±0.88 | P=0.09 (MW) |
| E-cadherin H-score | 166.52±68.92 | 182.43±65.09 | 167.60±61.69 | 13.89±39.55 | **P <0.001 (KW)** | 173.05±65.79 | **P<0.001 (MW)** |
| SSTR1 H-score | 21.18±44.35 | 10.45±15.56 | 3.36±6.56 | 3.91±9.11 | P = 0.13 (KW) | 14.14±31.73 | **P=0.029 (MW)** |
| SSTR2 H-score | 224.39±66.14 | 204.23±57.42 | 177.03±85.67 | 113.09±79.44 | **P <0.001 (KW)** | 209.04±67.16 | **P<0.001 (Student's t-test)** |
| SSTR3 H-score | 123.55±85.13 | 71.38±53.28 | 95.61±75.82 | 43.2±36.71 | **P <0.001 (KW)** | 98.39±75.31 | **P<0.001 (MW)** |
| SSTR5 H-score | 79.22±66.06 | 56.68±52.92 | 82.4±82.01 | 111.92±71.70 | **P = 0.012 (KW)** | 70.69±63.90 | **P<0.002 (MW)** |
| D2DR H-score | 34.49±42.13 | 43.51±33.32 | 56.15±52.73 | 30.05±32.31 | P = 0.22 (KW) | 41.43±40.73 | P=0.24 (MW) |
| AIP H-score | - | - | - | 227.21±37.88 |  | 243.39±38.99 | P=0.25 (Student's t-test) |
| Sex |  |  |  |  |  |  |  |
| Men | 17 | 15 | 5 | 19 |  | 37 |  |
| Women | 12 | 11 | 5 | 26 | P=0.47 (χ^2^) | 28 | P=0.19 (χ^2^) |
| Tumour size |  |  |  |  |  |  |  |
| Microtumours | 3 | 3 | 3 | 4 |  | 9 |  |
| Macrotumours | 23 | 22 | 7 | 35 |  | 52 |  |
| Giant tumours | 1 | 0 | 0 | 3 | P=0.47 (χ^2^) | 1 | P=0.29 (χ^2^) |
| Proliferativity (n=98) |  |  |  |  |  |  |  |
| Trouillas A | 21 | 17 | 7 | 22 |  | 45 |  |
| Trouillas B | 6 | 6 | 3 | 16 | P=0.34 (χ^2^) | 15 | P=0.12 (χ^2^) |
| p53 Expression (n=107) |  |  |  |  |  |  |  |
| Positive | 18 | 17 | 7 | 24 |  | 22 |  |
| Negative | 10 | 9 | 3 | 19 | P = 0.77 (χ^2^) | 42 | **P = 0.046 (χ^2^)** |
| Invasion (n=83) |  |  |  |  |  |  |  |
| Non-invasive (Knosp 0-2) | 14 | 18 | 6 | 17 |  | 38 |  |
| Invasive (Knosp 3 and 4) | 7 | 6 | 1 | 14 | P = 0.28 (χ^2^) | 14 | P=0.14 (χ^2^) |
| Trouillas grade (n=73) |  |  |  |  |  |  |  |
| 1a | 13 | 10 | 3 | 12 |  | 26 |  |
| 2a | 3 | 5 | 1 | 4 |  | 9 |  |
| 1b | 1 | 6 | 3 | 3 |  | 10 |  |
| 2b | 3 | 0 | 0 | 6 | P = 0.12 (χ^2^) | 3 | P=0.16 (χ^2^) |
